# Network Evolution Model-based prediction of tumor mutation burden from radiomic-clinical features in endometrial cancers

**DOI:** 10.1186/s12885-023-11118-4

**Published:** 2023-07-31

**Authors:** Qing Tan, Qian Wang, Suoqin Jin, Fuling Zhou, Xiufen Zou

**Affiliations:** 1 School of Mathematics and Statistics, Wuhan University, Wuhan, 430072 China; 2 Hubei Key Laboratory of Computational Science, Wuhan University, Wuhan, 430072 China; 3 Department of Hematology, Zhongnan Hospital of Wuhan University, Wuhan, 430072 China

**Keywords:** Endometrial cancer, Radiomic-clinical features, Tumor mutation burden, Network evolution model, Transfer information entropy

## Abstract

**Background:**

Endometrial Cancer (EC) is one of the most prevalent malignancies that affect the female population globally. In the context of immunotherapy, Tumor Mutation Burden (TMB) in the DNA polymerase epsilon (POLE) subtype of this cancer holds promise as a viable therapeutic target.

**Methods:**

We devised a method known as NEM-TIE to forecast the TMB status of patients with endometrial cancer. This approach utilized a combination of the Network Evolution Model, Transfer Information Entropy, Clique Percolation (CP) methodology, and Support Vector Machine (SVM) classification. To construct the Network Evolution Model, we employed an adjacency matrix that utilized transfer information entropy to assess the information gain between nodes of radiomic-clinical features. Subsequently, using the CP algorithm, we unearthed potentially pivotal modules in the Network Evolution Model. Finally, the SVM classifier extracted essential features from the module set.

**Results:**

Upon analyzing the importance of modules, we discovered that the dependence count energy in tumor volumes-of-interest holds immense significance in distinguishing TMB statuses among patients with endometrial cancer. Using the 13 radiomic-clinical features extracted via NEM-TIE, we demonstrated that the area under the receiver operating characteristic curve (AUROC) in the test set is 0.98 (95% confidence interval: 0.95–1.00), surpassing the performance of existing techniques such as the mRMR and Laplacian methods.

**Conclusions:**

Our study proposed the NEM-TIE method as a means to identify the TMB status of patients with endometrial cancer. The integration of radiomic-clinical data utilizing the NEM-TIE method may offer a novel technology for supplementary diagnosis.

**Supplementary Information:**

The online version contains supplementary material available at 10.1186/s12885-023-11118-4.

## Background

Endometrial cancer (EC) is a malignancy with a high incidence rate in women that can result in significant morbidity and mortality [1]. The conventional gold standard for prognostic factors, including tumor histology, grade, and International Federation of Gynecology and Obstetrics (FIGO) stage, is often associated with a high degree of observational error, making it challenging to accurately diagnose patients [2–4]. In the Cancer Genome Atlas (TCGA), four molecular subtypes of EC, namely, DNA polymerase epsilon (POLE), mismatch-repair deficient (MMR-D), copy-number low (CN-low), and copy-number high (CN-high) have been utilized to determine the prognosis for personalized treatment [5]. Additionally, Tumor Mutation Burden (TMB) is being evaluated as a potential immunotherapy target for POLE EC patients to assess the efficacy of PD-1 therapy [6]. However, the current gold standard for identifying the TMB status of EC patients is through pathological analysis and whole-exome sequencing (WES), which is relatively unstable and expensive, limiting the accuracy of prognostic analysis.

Radiomics is an emerging field that utilizes quantitative image features extracted from medical imaging data to enhance diagnostic, prognostic, and predictive accuracy [7]. Recent studies have shown that radiomics features can predict TMB status using machine learning methods in two main directions. The first direction involves traditional feature mining and classification. For instance, Harini et al. (2020) [8] used imaging and clinical data to predict microsatellite instability and high tumor mutation burden from contrast-enhanced computed tomography in EC patients, achieving a test set AUC of 0.87. The second direction utilizes deep learning frameworks to identify TMB status. He et al. (2020) [9] employed a 3D convolution kernel deep learning model to predict TMB status in non-small-cell lung cancer patients, achieving a test set AUC of 0.81. Although these studies perform well in TMB status identification, they still have certain limitations. The first method is insufficient in mining the relationships between features, while the second method based on deep learning has poor interpretability. Furthermore, these methods are challenging to apply for classification problems with small sample sizes and high dimensions, necessitating the consideration of the reliability of the classification results.

Integrated radiomic-clinical data of EC patients present challenges due to small sample size and high feature dimension. Traditional machine learning methods struggle to obtain satisfactory results in mining the correlation between TMB status and integrated radiomic-clinical data. Therefore, it is necessary to select the most excellent radiomic-clinical feature dimension before prediction analysis. Existing feature selection algorithms, such as the minimum-redundancy maximum-relevancy (mRMR)[10] algorithm and the Laplacian Score[11] method, lack balance in their influence between individuals and groups to the target. To overcome these limitations, we propose a novel algorithm based on Network Evolution Model, denoted as NEM-TIE, which effectively explores the correlation between integrated radiomic-clinical data of EC patients and TMB status. The proposed NEM-TIE is evaluated for its effectiveness.

## Methods

The methodology section includes two parts: data processing and model framework. In the data processing part, clinical and imaging data of EC patients from Harini's 2020 study were utilized, and the TMB status of EC patients was classified based on literature [12]. In the model framework part, the transfer information entropy was employed to establish the Evolution Network model, and the CP algorithm was used to detect differential modules within the network that were linked to TMB status in EC patients. Afterwards, the influence of differential modules on the identification of TMB status in EC patients was evaluated through statistical indicators. The radiomic-clinical features within the identified differential modules were considered as the predicted biomarker for distinguishing TMB statuses among patients. Below we described these two parts in detail.

### Data collection

The radiomic and clinical data were collected from Memorial Sloan Kettering Cancer Center from Harini’s research (2020) [8]. According to the eligibility criteria with histologic subtypes of EC and FIGO stages, 150 patients were selected and used for follow-up analysis. This cohort was randomly divided into three groups: the train group (*n* = 105, 70%), the test group (*n* = 30, 20%), and the validation group (*n* = 15, 10%). Pertinent clinical information was extracted by reviewing electronic medical records. The details of EC characteristics can be found in Table 1.

**Table 1:** Patient characteristics

	All	Train	Test	Validation
	*N* = 150	*N* = 105	*N* = 30	*N* = 15
**Median patient age, years**	63.6	63.5	65.2	61.2
**Histology, number**				
Endometrioid	64	46	11	7
Serous	33	21	7	5
Clear cell	11	8	3	0
Carcinosarcoma	29	23	6	0
Undifferentiated/dediffderentiated	6	5	1	0
Unclassified high-grade type	14	7	4	3
**Tumor grade, number**				
Well/moderately differentiated	114	79	26	9
Poorly differentiated	43	31	6	6
**Stage, number**				
Extra-uterine	89	61	20	8
Uterine-confined	68	49	12	7

### TMB interpretation and the standard with TMB-H and TMB-L

The Tumor Mutational Burden (TMB) corresponds to the count of genetic mutations present in a patient's tumor tissue. This metric can be calculated by employing MSK-IMPACT sequencing, which computes the number of nonsynonymous somatic mutations-per-megabase (mut/Mb) [12]. To differentiate between high and low TMB status, we have adopted a cut-off value of 15.5 mut/Mb. This threshold has been deemed significant in a clinical investigation on advanced solid tumor patients treated with Atezolizumab [8, 13].

### Radiomic data processing

During the tumor region segmentation phase, two radiologists, Yulia Lakhman and Josip Ninčević, who possess clinical expertise as outlined in Harini et al. (2020) [8], meticulously delineated all tumor margin information. The radiologists employed the Insight Segmentation and Registration Toolkit Segmentation platform (ITK-SNAP) to label the tumor VOI (volume of interest) within patients afflicted with endometrial cancers.

To guarantee the uniformity of texture feature extraction, all images underwent resampling to achieve 1 × 1x1 mm^3^ voxels via the utilization of ITK software. In conjunction with the tumor volume of interest (VOI), the adjacent peritumoral rim VOI was also scrutinized, in order to account for the effects of the surrounding milieu on the tumor VOI. Initially, an area 3 mm beyond the periphery of the tumor VOI was automatically generated, and the area which did not include the tumor VOI was subsequently designated as the peritumoral rim VOI.

Radiomic features are designed to assess the volumes-of-interest (VOI) of the tumor and the surrounding peritumoral rim VOI. In the present study, these features were computed utilizing the Computational Environment for Radiological Research (CERR, https://github.com/cerr/CERR/). A total of two hundred features were extracted from both the tumor VOI and peritumoral-rim VOI, with the former being encompassed by the latter.

### Construction of network evolution model (NEM-TIE) based on transfer information entropy

The Network Evolution Model is from a graph-based idea which means the new links depend on the local network structure [14]. The integrated radiomic‑clinical features are regarded as nodes of the graph G = (C, V). Moreover, the measure of the correlation between the different features is used as an edge to construct a network.

Edge connection conditions are defined based on information gain. The *p*_*x*_, *p*_*y*_ and *p*_*xy*_ represent the probability of Linear Discriminant Analysis (LDA) classification accuracy for feature nodes *X*, *Y* and their joint, respectively. The high value of *E* means there is the excellent information gain for the classification task with label *C* when *X* and *Y* exist simultaneously [15].

1 $$E\left( {X,Y|C} \right) = p_{xy} \log \left( {\frac{{p_{xy} }}{{p_{x} p_{y} }}} \right)$$

Furthermore, we normalized to obtain the adjacency matrix R in NEM-TIE in which each element *R*_*jk*_ is calculated as follows:

2 $$R_{jk} = N\left( {E\left( {X_{j} ,X_{k} |C_{m} } \right)} \right)$$

where *X*_*j*_ and *X*_*k*_ represent the *j*^th^ and *k*^th^ columns of the sample-feature matrix *X*_*mn*_. The function *N*(.) represents normalization of formula (1) and it makes that *R*_*jk*_ belongs to [0,1]. If *R*_*jk*_ is larger than threshold *T*, the feature nodes *X*_*j*_ and *X*_*k*_ have an edge, otherwise no edge between them.

### Linear discriminant analysis (LDA) method for refining modules in NEM

We first used clique percolation (CP) method [16] to calculate the set of all module structures in NEM-TIE for all samples, denoted as $CP\left( {R,T} \right)$ and their corresponding feature submatrix are denoted as $X_{ms}$ (*s* = 1,2,…, *D*, *D* is the total number of the modules). Further, Linear Discriminant Analysis (LDA) in machine learning was motivated to identify the optimal modules. For all $X_{ms}$ in $CP\left( {R,T} \right)$, the following values $Z_{{m{\text{s}}}}$ are calculated according to the rule in LDA

3 $$\begin{aligned} Z_{ms} = LDA\left( {X_{ms} } \right) = \frac{{\left\| {\left( {\mu_{s}^{ + } - \mu_{s}^{ - } } \right)\left( {\mu_{s}^{ + } - \mu_{s}^{ - } } \right)^{T} } \right\|}}{{\left\| {\frac{1}{{N_{1} - 1}}\sum\limits_{i = 1}^{{N_{1} }} {\left| {X_{is}^{ + } - \mu_{s}^{ + } } \right|^{2} } + \frac{1}{{N_{2} - 1}}\sum\limits_{j = 1}^{{N_{2} }} {\left| {X_{js}^{ - } - \mu_{s}^{ - } } \right|^{2} } } \right\|}} \hfill \\ s = 1,2,...,D \hfill \\ \end{aligned}$$

where the submatrix $X_{ms}$ divides into the *N*_*1*_ positive samples $X_{ms}^{ + }$ and *N*_*2*_ negative samples $X_{ms}^{ - }$. $\mu_{s}^{ + }$ represents the mean of $X_{ms}^{ + }$ and $\mu_{s}^{ - }$ is the mean of $X_{ms}^{ - }$. In specifically, $\mu_{s}^{ + } = \frac{1}{{N_{1} }}\sum\limits_{i = 1}^{{N_{1} }} {X_{is}^{ + } }$ and $\mu_{s}^{ - } = \frac{1}{{N_{2} }}\sum\limits_{j = 1}^{{N_{2} }} {X_{js}^{ - } }$.

#### Identifying the optimal feature set from the refined modules

Given the $m \times n$ sample feature matrix *X* and indicator label vector *C*_*m*_. For the classifier *F* and the selected feature submatrix $\bigcup\limits_{v = 1}^{D} {X_{mv} }$. the identification of optimal feature set $X_{ml}$ is converted into the following optimization problem.

4 $$\begin{aligned} \mathop {\min }\limits_{{X_{ml} }} \sum\limits_{i = 1}^{m} {\sum\limits_{s = 1}^{D} {\left| {F\left( {X_{is} } \right) - C_{i} } \right|} } \hfill \\ \begin{array}{*{20}c} {s.t.} & {X_{ml} \subset \bigcup\limits_{v = 1}^{D} {X_{mv} } .} \\ \end{array} \hfill \\ \end{aligned}$$

### Loss function for extracting the main feature combinations in optimized modules

In order to guarantee the training and testing accuracy, we used the following formula as loss function.

5 $$AUC = \alpha AUC_{train} + \beta AUC_{test}$$

where $AUC_{train}$ and $AUC_{test}$ are the training and testing accuracy, respectively. $\alpha$ and $\beta$ are the parameters can be adjusted. In this study, we used warp SVM to calculate the target AUC indicator and used PSO to obtain optimal threshold T.

The PSO algorithm is employed to fine-tune this hyperparameter. Specifically, the threshold T and CP method are utilized to generate the module set *X*_*ms*_. The combined features in *X*_*ms*_ are evaluated using the SVM classifier, and their classification performance is measured by the area under the curve (AUC). Subsequently, the PSO algorithm calculates the AUC-based loss function of the threshold T to obtain the optimal value.

### The algorithm workflow

**Fig. 1:**
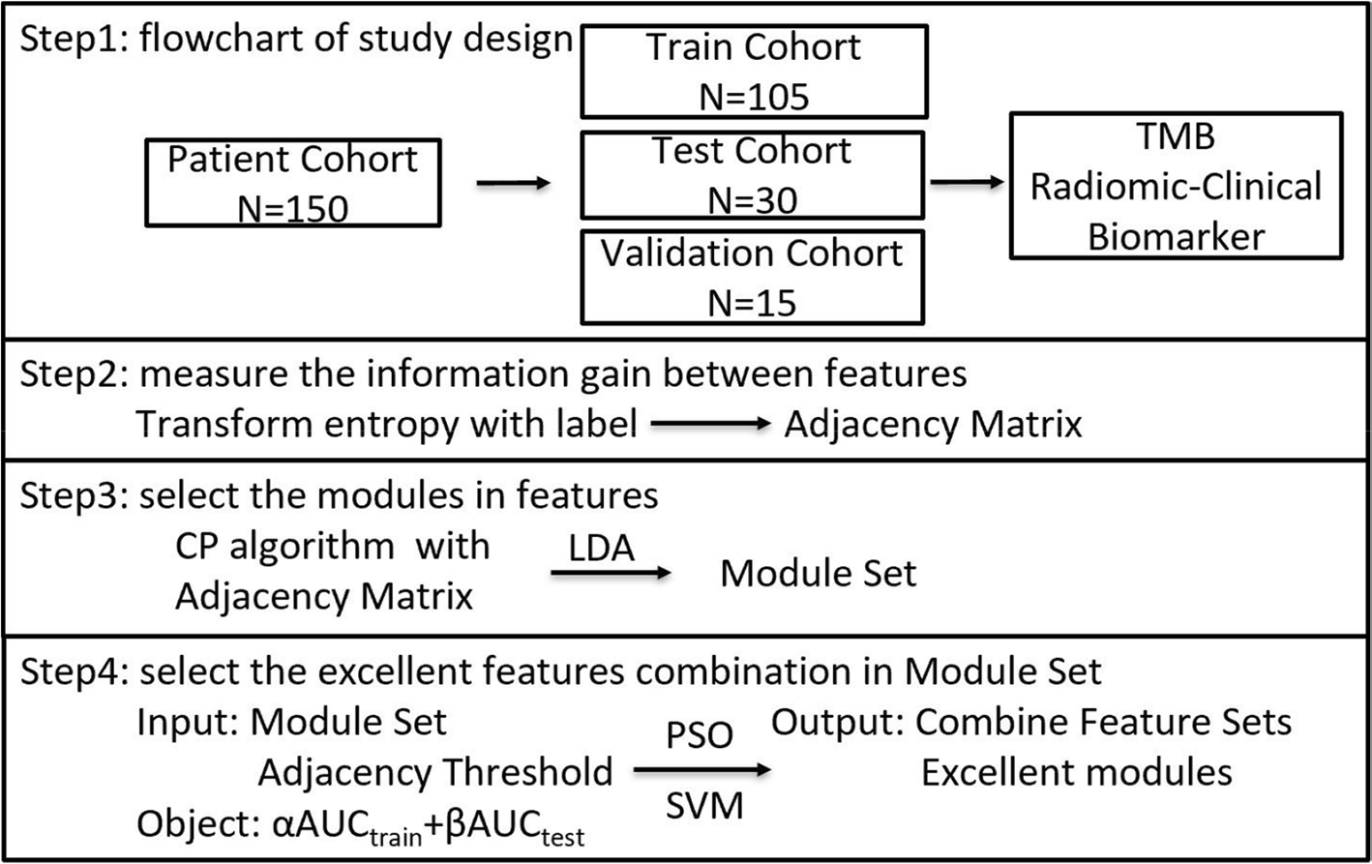
The main algorithm workflow in this study

To clearly elucidate the computational process, the workflow is depicted in Fig. 1, which includes four steps. Initially, the distribution of sample numbers was determined as the first step. The second step involved measuring the information gain between features by employing LDA and transform entropy. In the third step, the CP algorithm was utilized to calculate the module set with the help of an initial adjacency threshold. Finally, the PSO algorithm [17] and SVM [18] were implemented to optimize the adjacency threshold and acquire the combination of optimal features in module sets. The parameter setting consisted of two parts: the first part involved setting α = β = 1/2 in the object setting, while the second part consisted of setting a = 0.8, and c1 = c2 = 1.49445 in the PSO setting.

### Statistical analysis

Pearson's correlation test and positive false discovery rate estimation were employed to validate the correlation between Integrated radiomic‑clinical data and TMB status. Additionally, the two-sided t-test and the one-sided t-test were employed to validate the distinctions among different models, confirming their preference and significance. Moreover, AUC, sensitivity, and specificity were utilized as evaluation metrics for the classification model. The code implementation platform utilized in this study was Matlab 2020a, and the corresponding specific code files are provided in the supplementary materials.

## Results

### Overview of the proposed NEM-TIE for identifying Endometrial Cancer TMB status

**Fig. 2:**
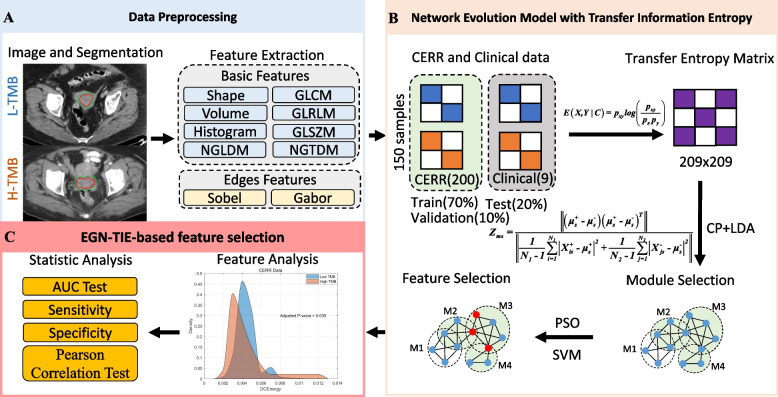
The framework of the proposed NEM-TIE in this study. **A**. For data preprocessing part, the CERR data were extracted by Basic Features and Edges Features form CT images. **B**. For NEM-TIE model part, there are three sub-steps. 1) The transfer entropy matrix was calculated by integrating clinical data and CERR data. 2) NEM was obtained based on transfer entropy matrix. 3) The excellent modules in NEM-TIE were selected by using CP and LDA and good features in excellent modules were filtered by using PSO and SVM. **C**. For feature selection part, feature analysis and statistical analysis were performed on the selected features and modules

The proposed NEM-TIE framework is illustrated in Fig. 2. It aims to identify the TMB status in EC patients by extracting superior modules and features. To achieve this goal, NEM-TIE integrates clinical and CERR data as inputs (Fig. 2A). The CERR data are subjected to eight basic filters and two edge filters to extract texture and edge information about tumor VOI and peritumoral-rim VOI. The network evolution model is constructed based on transfer information entropy using the minimum adjacency threshold on the nodes of radiomic-clinical data. The transfer entropy matrix corresponds to the network evolution model, and the CP and LDA methods are employed to select modules that perform well in formula 3. In addition, PSO and SVM are used to filter out excellent features (Fig. 2B). Finally, for the selected features and modules, feature analysis and statistical analysis, such as AUC test and Pearson correlation test, are carried out (Fig. 2C).

### NEM-TIE method accurately identifies TMB‑H tumors by integrating radiomic‑clinical data

**Fig. 3:**
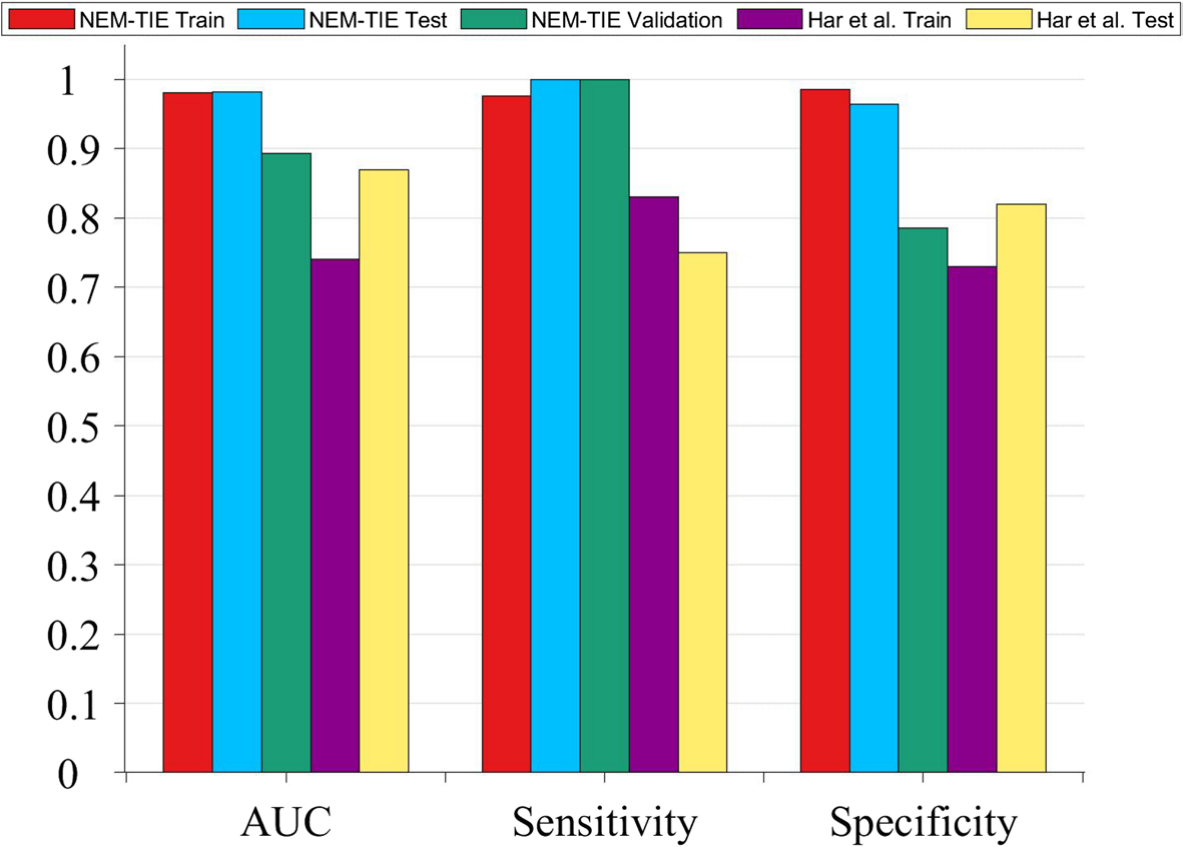
Comparison of NEM-TIE and the other reference in the same dataset

The performance of the NEM-TIE method to distinguish TMB status from TMB-H and TMB-L EC patients was summarized in Table 2. Compared with a previous study [8], we found our model is competitive which has achieved the AUC of 0.98 (95% confidence interval: 0.95–1.00) for the test dataset and 0.89 (95% CI: 0.46–1.00) for the validation dataset (Fig. 3).

**Table 2:** High TMB vs Low TMB results (with 95% CI) with NEM-TIE method

	AUC	Sensitivity	Specificity
Train	0.9807 (0.9509–1.0000)	0.9762 (0.9477–1.0000)	0.9853 (0.9628–1.0000)
Test	0.9821 (0.8890–1.0000)	1.0000 (1.0000–1.0000)	0.9643 (0.9000–1.0000)
Validation	0.8929 (0.4602–1.0000)	1.0000 (1.0000–1.0000)	0.7857 (0.5781–0.9934)

### NEM-TIE method exhibits better performance in comparison with existing methods

**Fig. 4:**
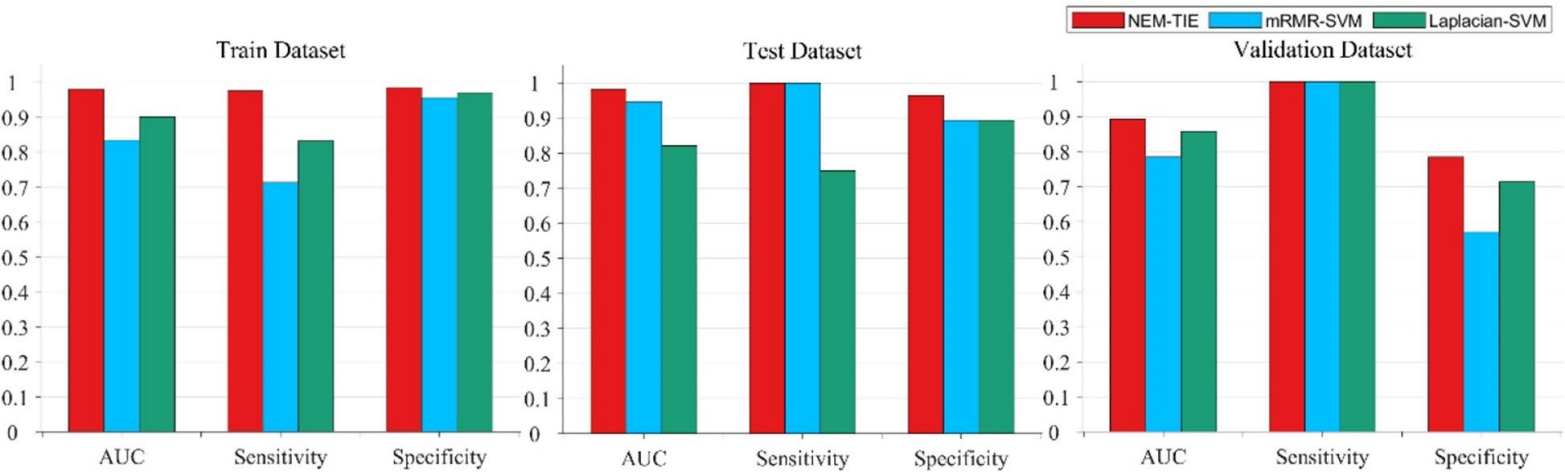
Comparison of NEM-TIE against other two methods

**Fig. 5:**
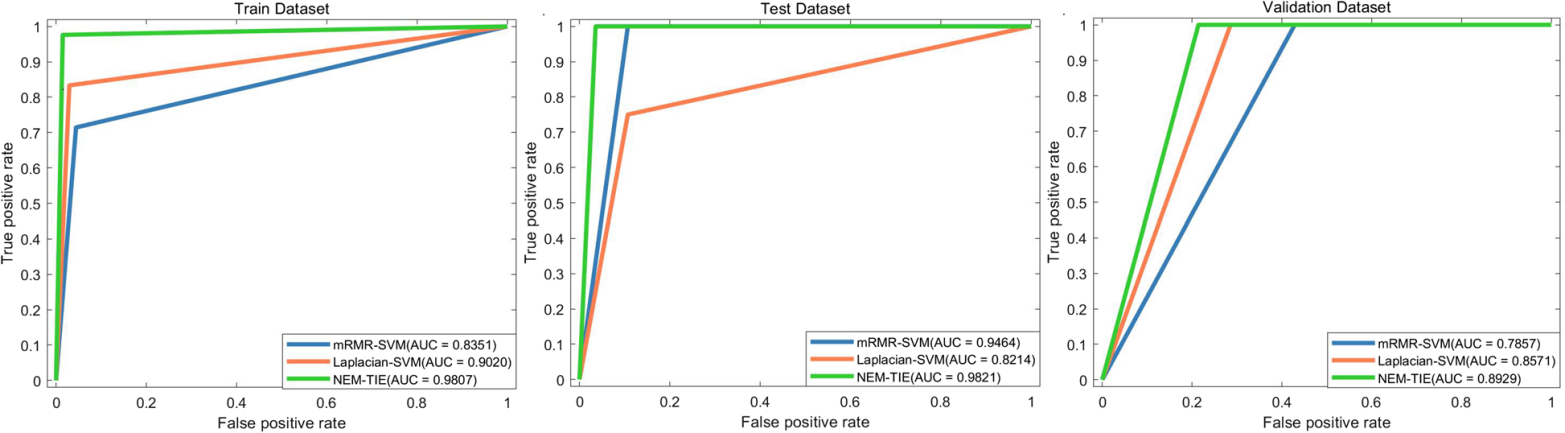
ROC curves of NEM-TIE in predicting TMB status against other two methods

The mRMR method and the Laplacian Score method are widely used in the feature selection. The performance of the mRMR-SVM method and Laplacian-SVM method to distinguish TMB status with EC patients are shown in Tables 3 and 4. Upon comparing the results presented in Table 2 with those in Tables 3 and 4, it is evident that the NEM-TIE method consistently exhibits superior performance, in terms of AUC, across the three groups for distinguishing the TMB status of EC patients. Both the histogram curve (Fig. 4) and the ROC curve (Fig. 5) consistently demonstrate that the NEM-TIE method exhibits superior discriminative power when compared to the mRMR-SVM and Laplacian-SVM methods. These results showed that NEM-TIE method consistently performs better than these two methods in identifying TMB status in EC patients.

**Table 3:** High TMB vs Low TMB results (with 95% CI) with mRMR-SVM method

	AUC	Sensitivity	Specificity
Train	0.8351 (0.7520–0.9182)	0.7143 (0.6299–0.7987)	0.9559 (0.9175–0.9943)
Test	0.9461 (0.7888–1.0000)	1.0000 (1.0000–1.0000)	0.8929 (0.7857–1.0000)
Validation	0.7857 (0.2285–1.0000)	1.0000 (1.0000–1.0000)	0.5714 (0.3210–0.8219)

**Table 4:** High TMB vs Low TMB results (with 95% CI) with Laplacian-SVM method

	AUC	Sensitivity	Specificity
Train	0.9020 (0.8361–0.9678)	0.8333 (0.7637–0.9030)	0.9706 (0.9390–1.0000)
Test	0.8241 (0.5595–1.0000)	0.7500 (0.6000–0.9000)	0.8929 (0.7857–1.0000)
Validation	0.8571 (0.3722–1.0000)	1.0000 (1.0000–1.0000)	0.7143 (0.4857–0.9429)

In order to ensure robustness and reliability of the results, we randomly sampled the data 10 times and using a ratio of 7:2:1 for the training set, test set, and validation set. By conducting a Two-Sided t-test, we have quantitatively evaluated the difference between the NEM-TIE method and other methods based on the AUC of test set. The significant differences with 95% CI observed between the NEM-TIE method and both the Laplacian-SVM method (p = 0.0018) and the mRMR-SVM method (*p* = 0.0005), indicate the superior predictive performance of the NEM-TIE method in predicting the TMB status of endometrial cancer. Additionally, the use of a One-Sided t-test to compare the NEM-TIE method with the method described in reference [8] provides further evidence of the NEM-TIE method's superior performance on the test set with a 95% CI (*p* = 0.0053). These statistical analyses strengthen the findings and demonstrate the effectiveness of the NEM-TIE method in predicting the TMB status in endometrial cancer when compared to other methods.

### NEM-TIE revealed important features and modules discriminating high and low TMB

**Fig. 6:**
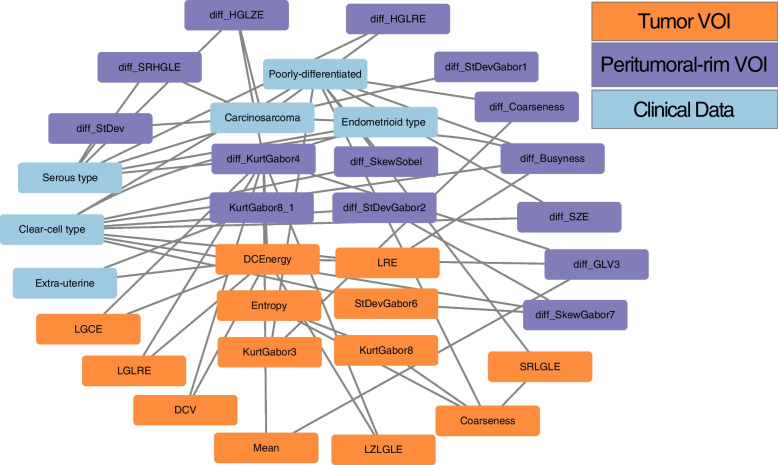
NEM-TIE selected modules network

The NEM-TIE method identified 13 features that are crucial for classification. All the extracted clinical features passed the Pearson correlation test and had a positive false discovery rate for the multiple hypothesis test (*p* <  = 0.001), as shown in Table 5. These 13 selected features and 22 correlation modules were visualized in a network correlation map presented in Fig. 6. The three colors in the figure represent the radiomic features of the Tumor VOI, the radiomic features of the Peritumoral-rim VOI, and the clinical data. The middle 13 critical features are feature nodes with relatively high degrees, which cover almost all 33 features associated with 22 modules. By combining the information in Table 2 and Fig. 3, the algorithm in this study eventually extracts 13 features that can effectively distinguish the high TMB and low TMB status of EC patients. These features provide novel and effective radiomic-clinical biomarkers for auxiliary diagnosis.

**Table 5:** NEM-TIE selected features

Selected Features	Adjusted p-value	Degree
**Endometrioid type**	** < 0.001**	**3**
DCEnergy	0.035	8
Entropy	0.120	2
StDevGabor6	0.008	2
KurtGabor8	0.110	2
diff_SkewSobel	0.096	2
**Poorly-differentiated**	** < 0.001**	**8**
diff_KurtGabor4	0.100	10
KurtGabor8_1	0.063	2
LRE	0.112	2
**Carcinosarcoma**	** < 0.001**	**7**
diff_StDevGabor2	0.002	2
KurtGabor3	0.098	2

The feature set identified encompassed six tumor VOI features, four peritumoral-rim VOI features, and three clinical features. Among the tumor VOI features, the Dependence Count Energy (DCEnergy, Image Biomarker Standardization Initiative, IBSI [19] 3.11.17) feature was derived from the *Neighborhood Gray Level Dependence Matrix* (NGLDM), which measures textural variations. The Entropy (IBSI 3.6.12) feature was obtained from *Intensity Histogram Features*, which quantifies Shannon entropy within the image. The Long Run Emphasis (LRE, IBSI 3.7.2) feature was derived from the *Gray Level Run Length Matrix* (GLRLM) and evaluates the distribution of discretized grey levels. Additionally, the StDevGabor6, KurtGabor3, and KurtGabor8 features were derived from Gabor wavelet filters and can be used to measure image edges.

**Fig. 7:**
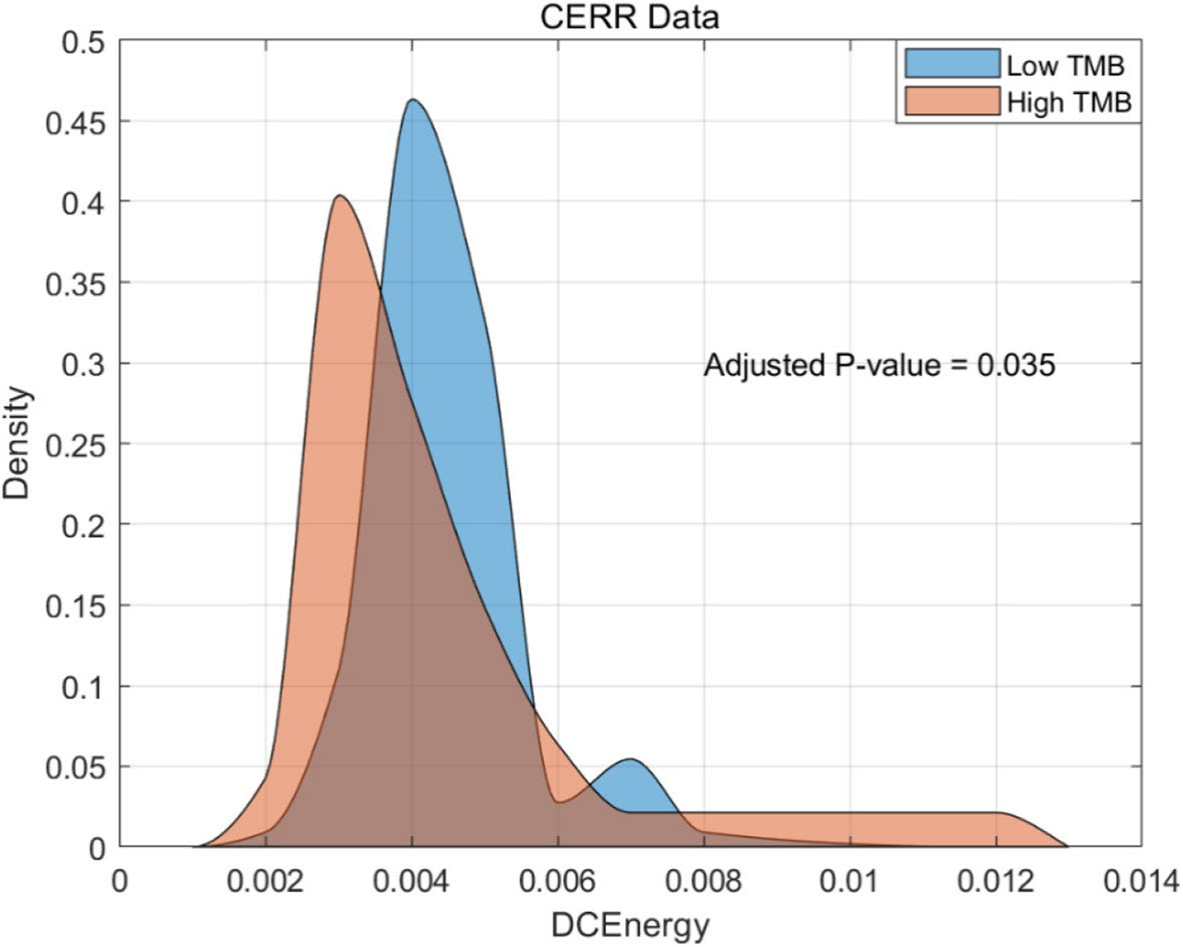
The distribution of CERR feature between high and low TMB

**Fig. 8:**
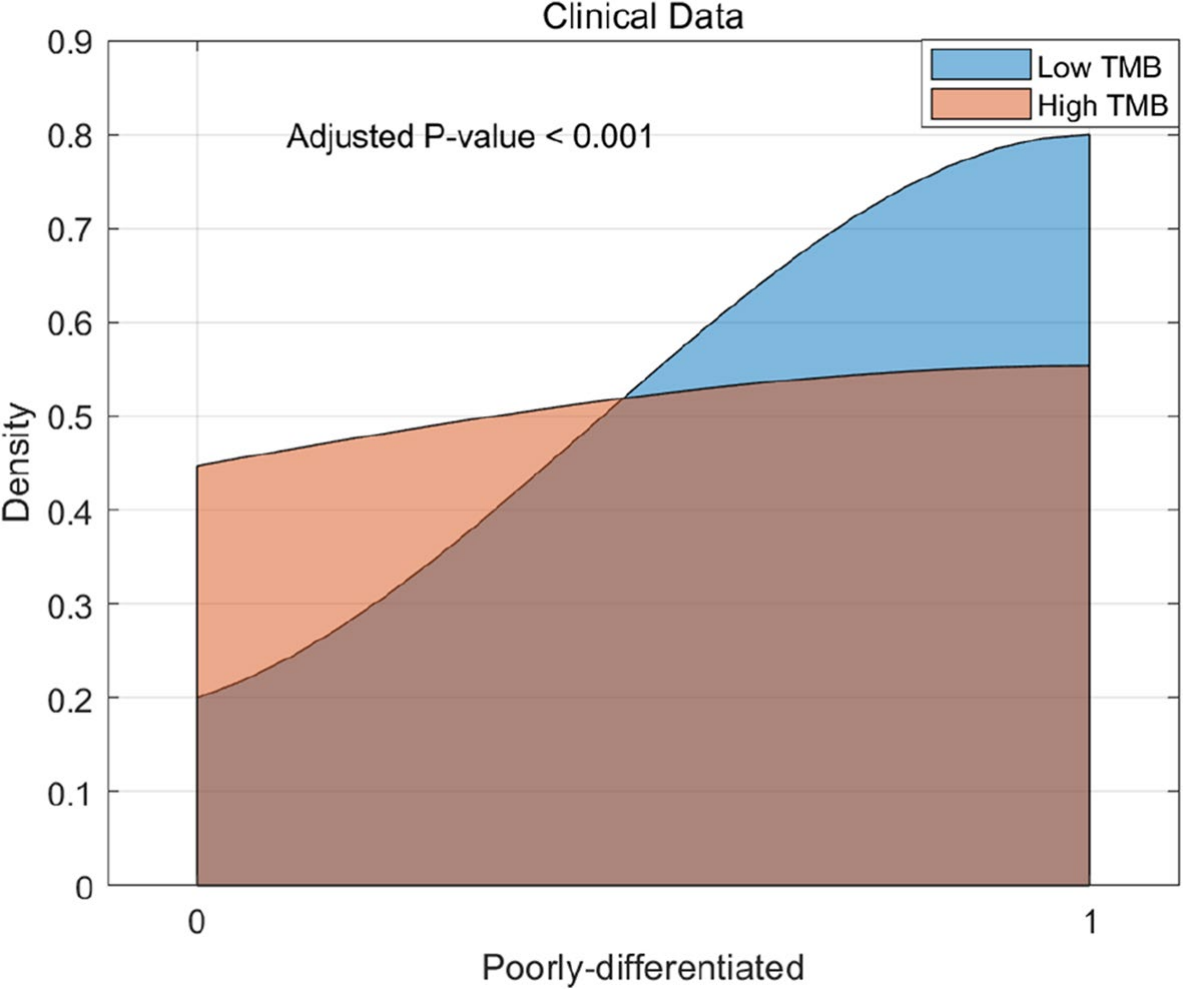
The distribution of Clinical feature between high and low TMB

Regarding the Peritumoral-rim VOI features, the four features, namely Sobel, KurtGabor4, KurtGabor8, and StDevGabor2, were derived from Gabor and Sobel filters and can also be utilized to measure image edges. As for the clinical features, Poorly-differentiated refers to the FIGO Grade 3 stage in EC patients [20], while Endometrioid type and Carcinosarcoma represent distinct tumor histology types. Interestingly, differences between TMB-L and TMB-H groups were observed in both the CERR and clinical features. Probability distribution curves were used to analyze the CERR feature DCEnergy and the clinical feature Poorly-differentiated in Figs. 7 and 8, respectively. The results revealed that the TMB-L group demonstrated superior performance in both DCEnergy and Poorly-differentiated features.

**Fig. 9:**
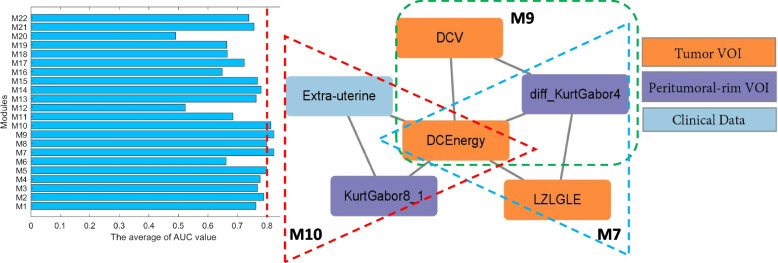
The result of modules analysis by using NEM-TIE method

The NEM-TIE algorithm extracted 22 modules, and their effects on TMB status in EC patients were analyzed separately. The features of each module were classified using SVM, and the results are presented in Table 6. By evaluating the average AUC performance of each module, three significant modules, namely modules 7, 9, and 10, were identified. The average AUC values for these three modules were all above 0.8, suggesting that they hold substantial value in distinguishing TMB status in EC patients. When we constructed a network based on these three modules, we discovered that the DCEnergy texture feature of the tumor VOI was at the core of the selected modules. This observation provides further evidence that DCEnergy is of vital importance for identifying TMB status in EC patients (Fig. 9).

**Table 6:** Module-based feature analysis

Module	Features			AUC_1_	AUC_2_	AUC_3_	AUC_4_
1	Mean	DCEnergy		0.7518	0.6071	0.9286	0.7625
	diff_GLV3	diff_KurtGabor4					
2	diff_HGLZE	diff_StDevGabor1		0.8512	0.8036	0.7143	0.7897
	Carcinosarcoma	Serous type					
3	Entropy	Coarseness	KurtGabor8	0.5896	0.7143	1.0000	0.7680
4	LRE	diff_Busyness	Clear-cell type	0.6894	0.7143	0.9286	0.7774
5	LGLRE	DCEnergy	diff_KurtGabor4	0.7370	0.7321	0.9286	0.7993
6	SRLGLE	Coarseness	Poorly-differentiated	0.7349	0.5000	0.7500	0.6616
7	**LZLGLE**	**DCEnergy**	**diff_KurtGabor4**	**0.7206**	**0.7857**	**0.9643**	**0.8235**
8	LGCE	DCEnergy	diff_KurtGabor4	0.7370	0.7321	0.9286	0.7993
9	**DCV**	**DCEnergy**	**diff_KurtGabor4**	**0.7206**	**0.7857**	**0.9643**	**0.8235**
10	**DCEnergy**	**KurtGabor8_1**	**Extra-uterine**	**0.6894**	**0.7857**	**0.9643**	**0.8131**
11	KurtGabor3	diff_Coarseness	Poorly-differentiated	0.6618	0.5357	0.8571	0.6849
12	StDevGabor6	diff_SkewGabor7	Clear-cell type	0.6043	0.4643	0.5000	0.5229
13	diff_StDev	Carcinosarcoma	Serous type	0.7899	0.6786	0.8214	0.7633
14	diff_HGLRE	Carcinosarcoma	Serous type	0.8218	0.8036	0.7143	0.7799
15	diff_SRHGLE	Carcinosarcoma	Serous type	0.8218	0.8036	0.6786	0.7680
16	diff_SZE	Clear-cell type	Poorly-differentiated	0.6425	0.5179	0.7857	0.6487
17	diff_HGLZE	diff_KurtGabor4	Serous type	0.5970	0.5714	1.0000	0.7228
18	diff_Busyness	Clear-cell type	Poorly-differentiated	0.6765	0.5000	0.8214	0.6660
19	diff_SkewSobel	Clear-cell type	Poorly-differentiated	0.6544	0.5179	0.8214	0.6646
20	diff_StDevGabor2	diff_SkewGabor7	Clear-cell type	0.6877	0.3929	0.3929	0.4911
21	diff_KurtGabor4	Endometrioid type	Serous type	0.7514	0.7321	0.7857	0.7564
22	Carcinosarcoma	Endometrioid type	Serous type	0.8043	0.7321	0.6786	0.7383

AUC_1_, AUC_2_, and AUC_3_ respectively represent the results of the training set, test set, and validation set. AUC_4_ is the average value of AUC_1_, AUC_2_ and AUC_3_

## Discussion

TMB serves as a significant biomarker and finds extensive application in immunotargeted therapy, particularly in the context of endometrial cancer [6]. In recent years, biomarker evaluation methods have been widely employed to address quantitative aspects in the field of bioinformatics. For instance, Nguyen Quoc Khanh Le et al. [21] proposed an XGBoosting-based model for recognizing Kruppel-like factors proteins in 2021, while Luu Ho Thanh Lam et al. [22] proposed the SMOTE-XGBoosting model for classifying low-grade glioma subtypes in 2022. In our study, we adopted a module-based evaluation method to assess the TMB status of patients with EC. Our study integrated clinical features and radiomic features using the NEM-TIE method to differentiate TMB status in EC patients. The AUROC of the NEM-TIE method in the test set was 0.98 (95%CI 0.95–1.00), which outperformed the Laplacian method and mRMR method in terms of performance. We have also evaluated the performance of one deep learning method called the deep stacked autoencoder network (SAE) [23], for predicting the TMB status using radiomic-clinical data of EC patients. By conducting 10 randomly sampled data experiments and using the two-sided t-test, we compared the performance of the NEM-TIE (0.98, 95%CI: 0.95–1.00) and the SAE (0.73, 95%CI: 0.46–1.00) in terms of the AUC performance on the test set. The results of our experiments showed that the NEM-TIE method outperformed the SAE method significantly in terms of predictive effect with a 95% CI (*p* < 0.0001). This indicates that the NEM-TIE method is more effective than the SAE method for predicting the TMB status in endometrial cancer using radiomic-clinical data. The poor performance of deep learning methods like SAE is likely due to the lack of an extensive number of labeled samples and the absence of model interpretability [24].

The Dependence count energy (DCEnergy) feature is a measure of the overall texture coarseness of an image. It is strongly associated with the second moment values of pixels that continuously change. Our findings reveal that, in comparison to TMB-H status, EC patients with TMB-L status exhibit higher average DCEnergy feature values. This phenomenon suggests that overall high DCEnergy in CT images, corresponding to the Tumor VOI of EC patients, is inversely related to TMB-H status.

The poorly-differentiated feature is a subtype of EC that is classified as grade 3 by FIGO. Tumors in EC patients with advanced FIGO are known to be aggressive and resistant to drugs [20]. Our findings reveal that, in comparison to TMB-H status, almost 80% of EC patients with TMB-L status are of the poorly-differentiated subtype. This phenomenon suggests that EC patients with TMB-L status are more likely to have aggressive and drug-resistant tumors due to their poorly-differentiated subtype.

Coarseness, as an important image feature derived from mathematical fractals, captures the repetition of simple image rules and is closely associated with the homogeneity of the Gray-Level Co-occurrence Matrix (GLCM). High coarseness implies low GLCM homogeneity, indicating a more heterogeneous texture pattern in the image [25]. The relevance of GLCM homogeneity in cancer research has been demonstrated in several studies. For instance, Shen et al. (2017) [26] found that GLCM homogeneity was an independent predictor of pelvic lymph node metastasis in patients with cervical cancer. By combining GLCM homogeneity with standardized uptake value (SUV) values, they were able to assess the risk of pelvic lymph node metastasis. Similarly, Yu et al. (2017) [27] reported that GLCM homogeneity played a significant role in risk stratification for stage I non-small cell lung cancer. They observed a significant correlation between GLCM homogeneity and overall survival, even after adjusting for factors such as age, tumor volume, and histological type. These studies highlight the importance of GLCM homogeneity, including its association with coarseness, in understanding tumor characteristics and predicting clinical outcomes in various types of cancer. By analyzing texture features derived from the coarseness, researchers can gain insights into the heterogeneity and homogeneity patterns within tumor images, enabling improved risk assessment and prognostic evaluation.

Our study demonstrated that the NEM-TIE method is effective in selecting the important modules and features that are relevant to the classification tasks. These extracted features were able to accurately predict the TMB status of EC patients, providing a non-invasive method for auxiliary diagnosis.

The evaluation method based on transfer information entropy and network modules indeed sets our approach apart from others. By utilizing transfer information entropy, we are able to capture the information gain between features within a module, thereby enhancing the evaluation of features. This approach takes into account the interactions between associated features, which is known to be more effective for classification tasks in machine learning than considering individual features in isolation. The incorporation of network modules in our method allows for a more comprehensive analysis of the relationships and dependencies among features, leading to improved predictive performance. These distinctive features contribute to the superior performance of our method compared to similar approaches. By utilizing transfer information entropy and network modules, our method demonstrates its capability to effectively extract relevant information and capture complex feature interactions, ultimately enhancing the prediction of TMB status in endometrial cancer.

It is important to acknowledge the limitations of the proposed method in the study. One limitation is that the radiomic features were extracted from a relatively small number of endometrial cancer patients (150 participants) and were based on labels and CERR radiomics signatures from previous studies. The use of a larger and more diverse dataset could potentially lead to more robust and generalizable results. A better model could be also benefited from the increasing number of patients. Additionally, the unified threshold of 15.5 mut/Mb used to distinguish TMB status may not be applicable to all subtypes of EC patients, and future studies could investigate subtype-specific thresholds. Finally, while some discussion is given in the study, the biological interpretation behind the prediction of TMB status in patients with endometrial cancer by radiomic-clinical data could be better evaluated by using other types of data.

## Conclusion

To summarize, our proposed NEM-TIE method has shown promising results in the non-invasive prediction of TMB status in EC patients by integrating clinical and radiomic features. Moreover, this method can be applied to analyze the relationship between feature module mining and biological indicators, allowing for further insights into the underlying mechanisms of TMB status in EC patients.

## Supplementary Information

**Additional file 1.**

## Data Availability

The support data with Endometrial cancer patients about this study is available at website https://github.com/harveerar/SciRepEndometrial2020.

## Abbreviation

EC: Endometrial cancer
TMB: Tumor mutation burden
POLE: DNA polymerase epsilon
CP: Clique percolation
VOI: Volumes-of-interest
NEM-TIE: Network evolution model-transfer information entropy
FIGO: International federation of gynecology and obstetrics
TCGA: The cancer genome atlas
MMR-D: Mismatch-repair deficient
CN-low: Copy-number low
CN-high: Copy-number high
PD-1: Programmed death-1
WES: Whole-exome sequencing
mRMR: Max-Relevance and min-Redundancy
SVM: Support vector machine
AUC: Area under the receiver operating characteristic curve
CERR: Computational environment for radiological research
LDA: Linear discriminant analysis
PSO: Particle swarm optimization
CI: Confidence interval
TMB‑H: High Tumor Mutation Burden
TMB‑L: Low Tumor Mutation Burden
ISBI: Image Biomarker Standardisation Initiative
DCEnergy: Dependence count energy
NGLDM: Neighborhood gray level dependence matrix
LRE: Long run emphasis
GLRLM: Gray level run length matrix
GLCM: Co-occurrence Matrix
